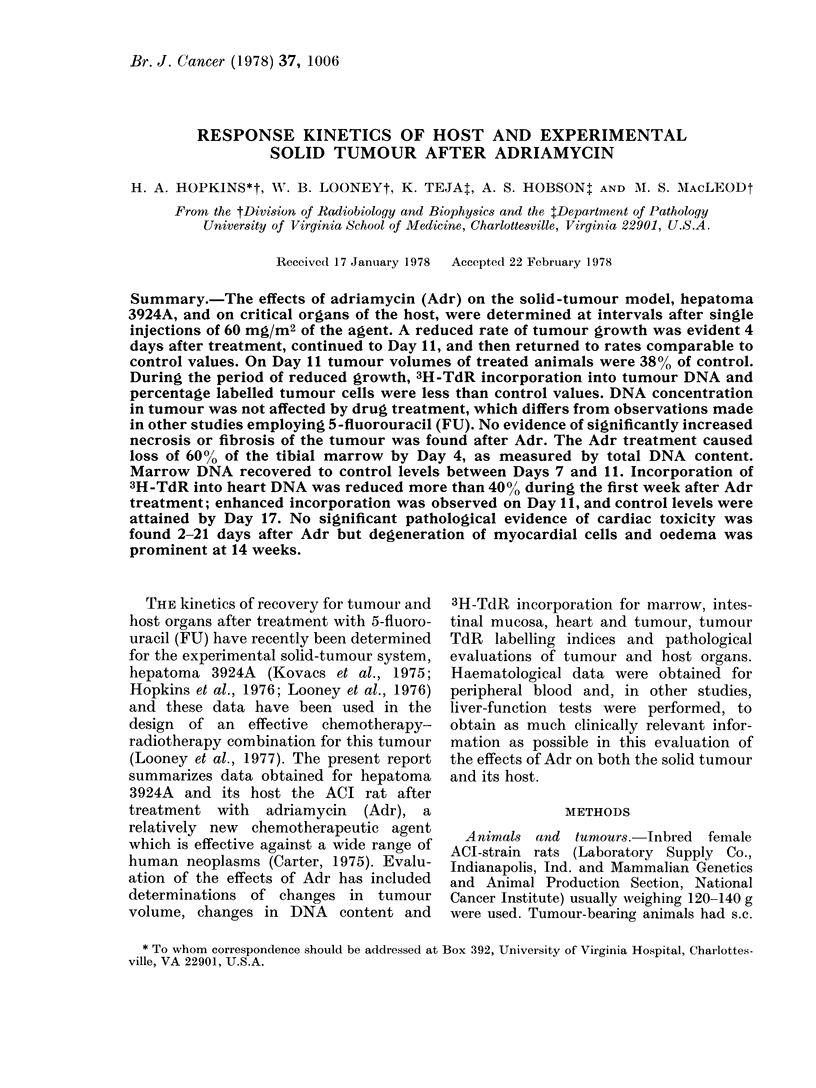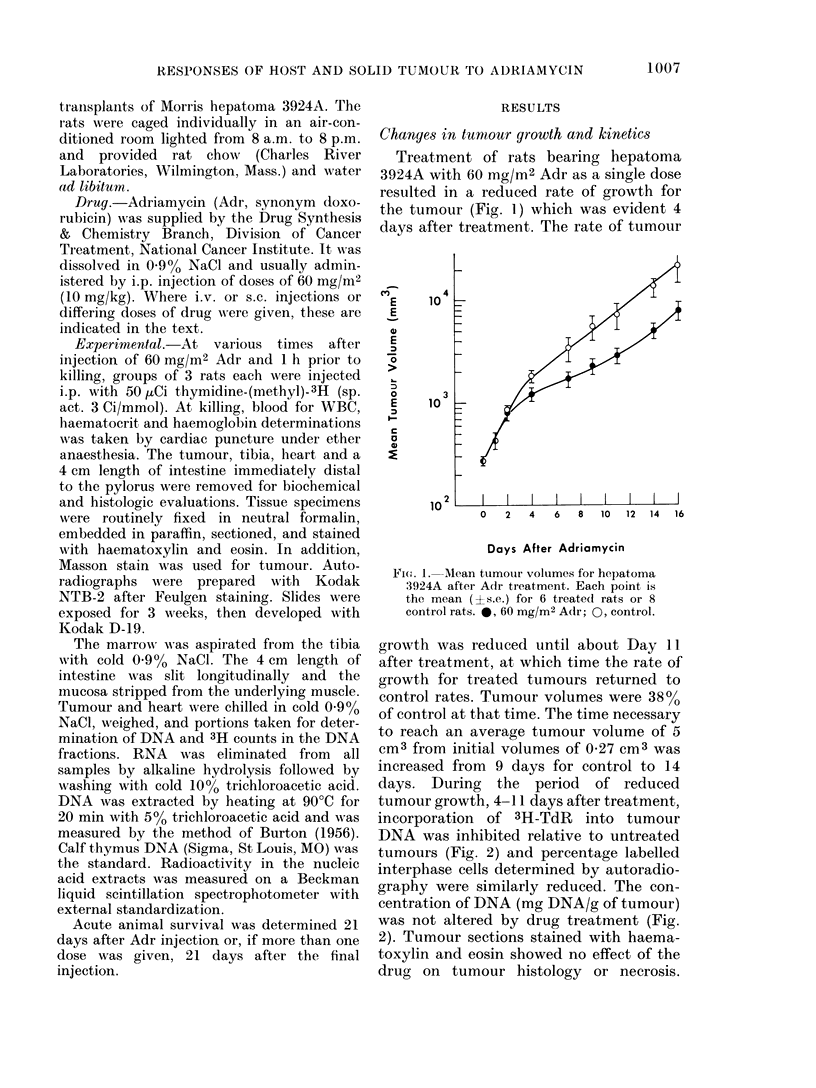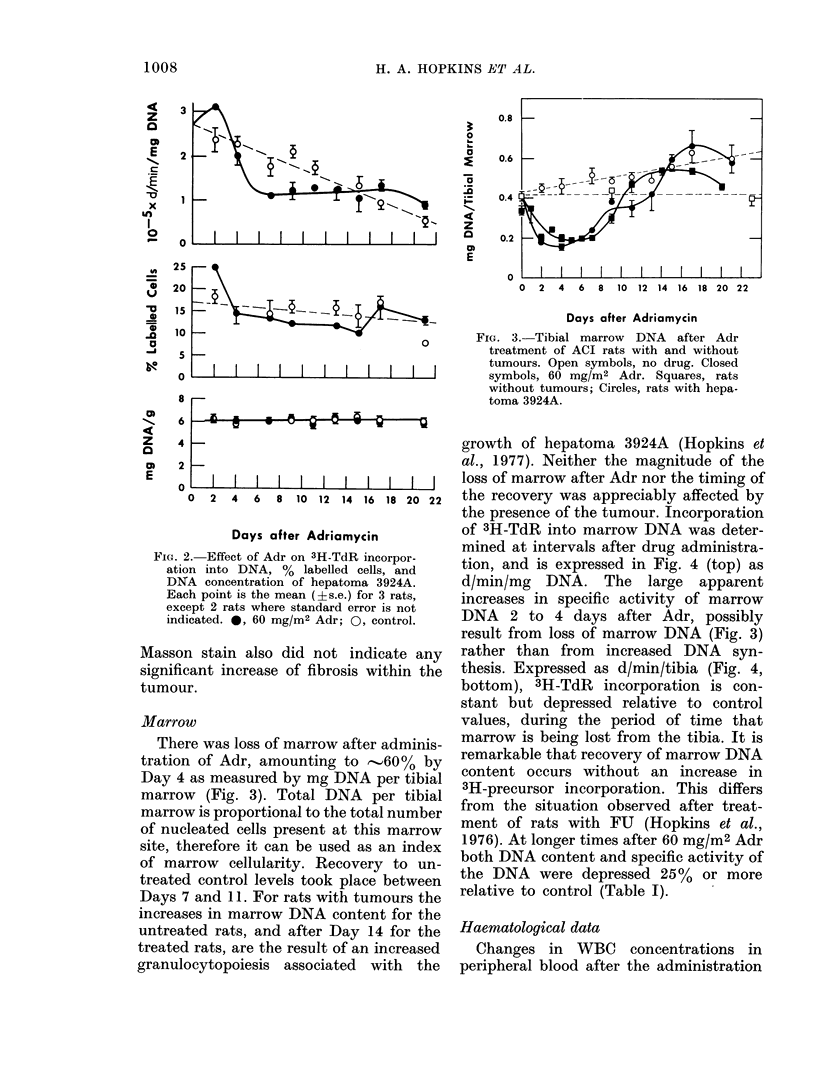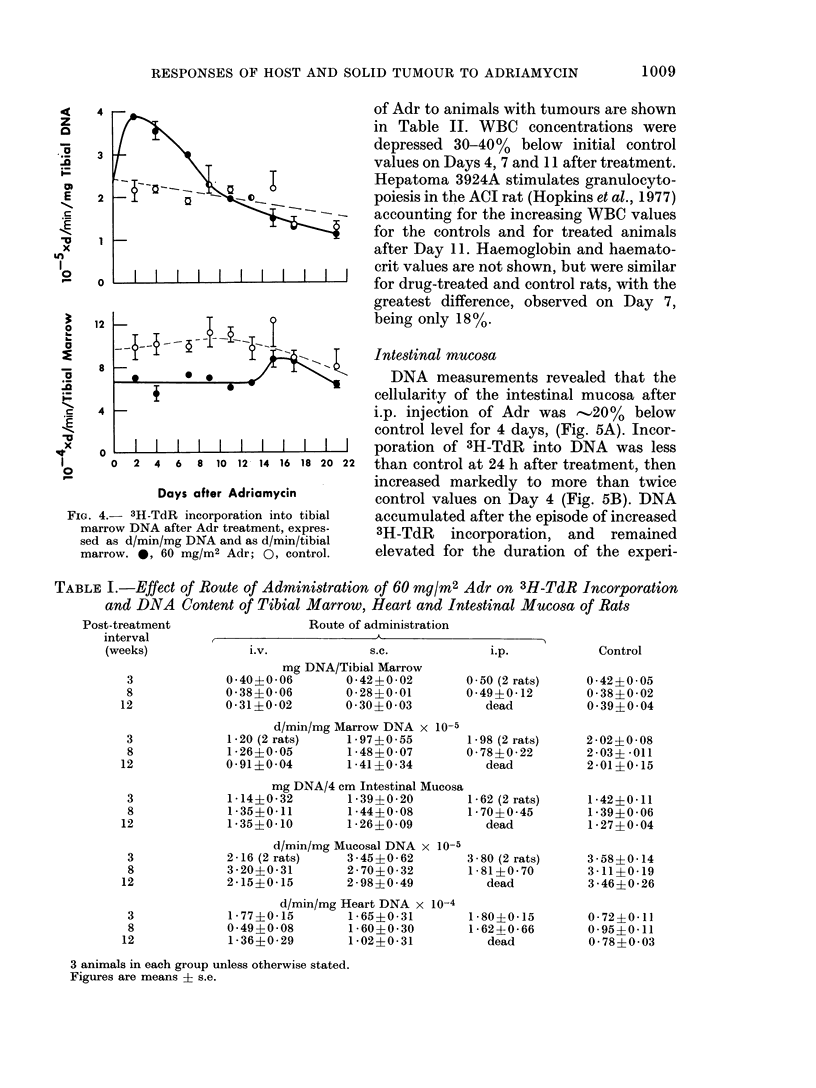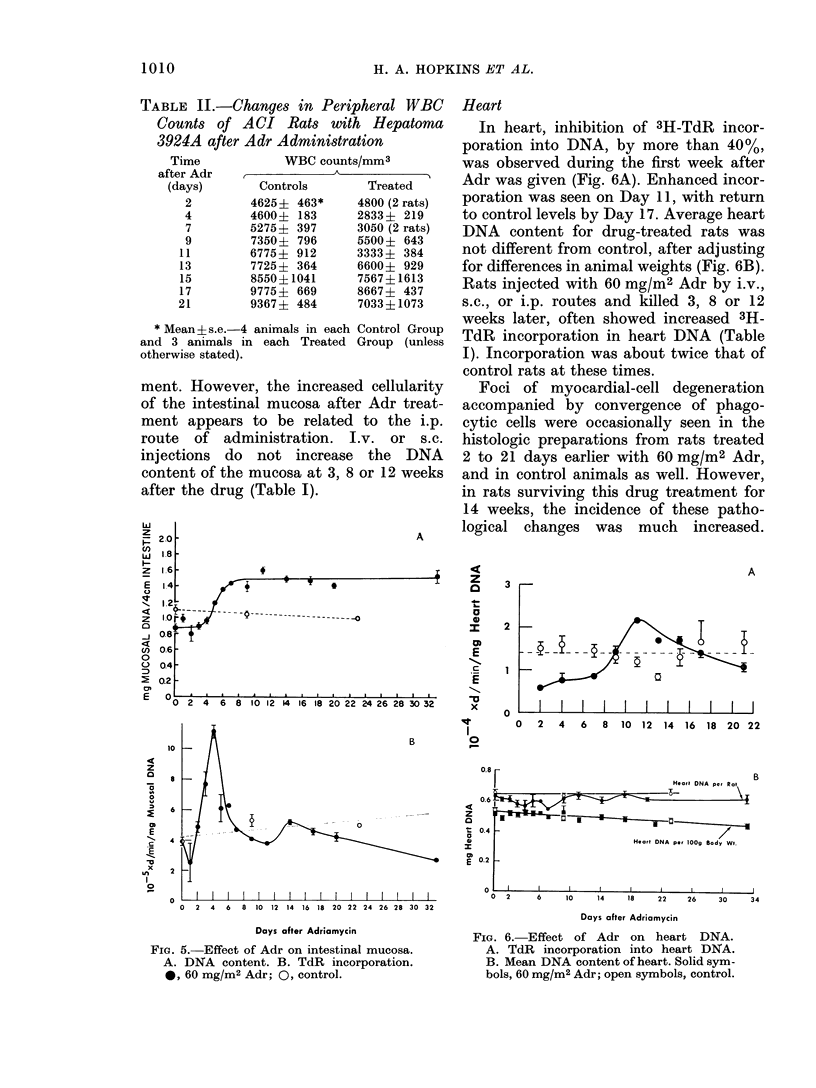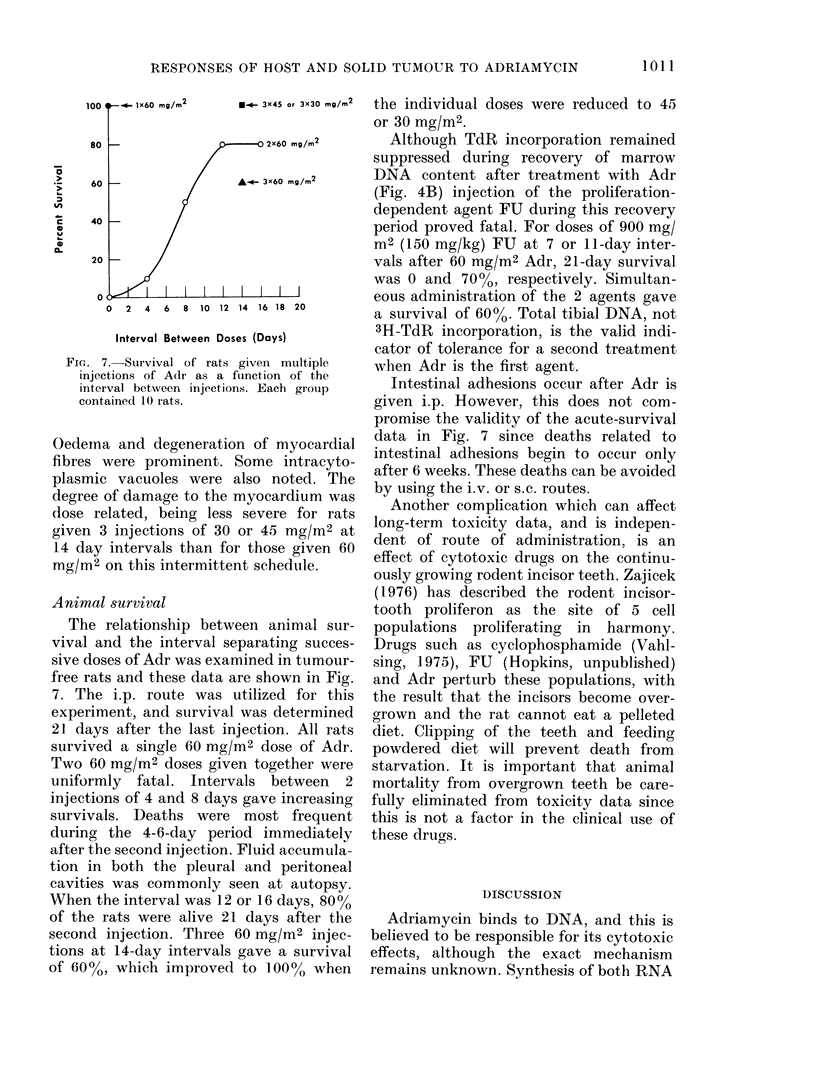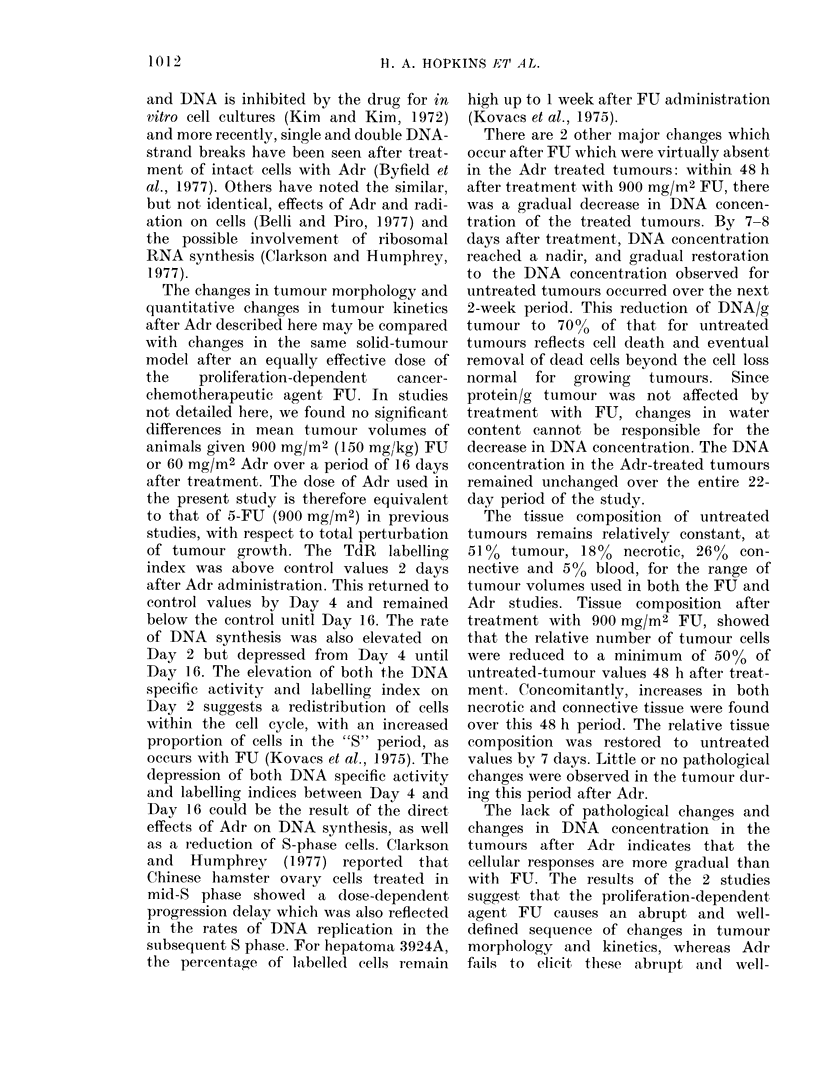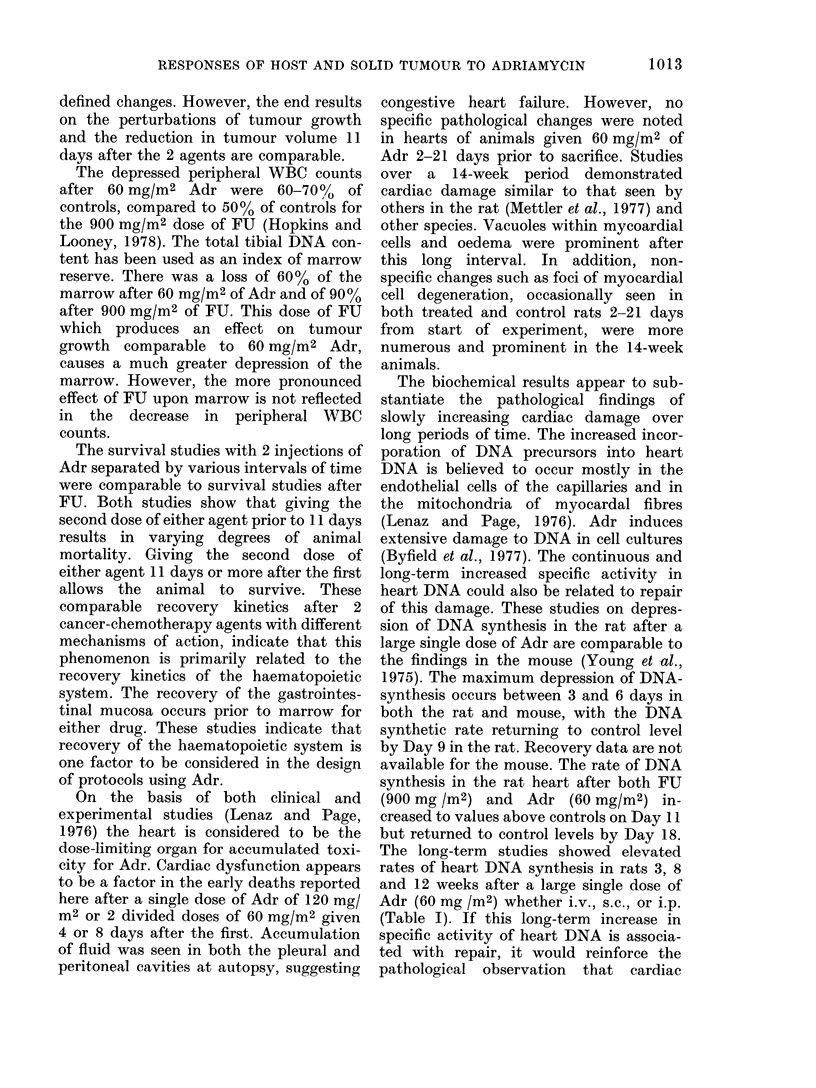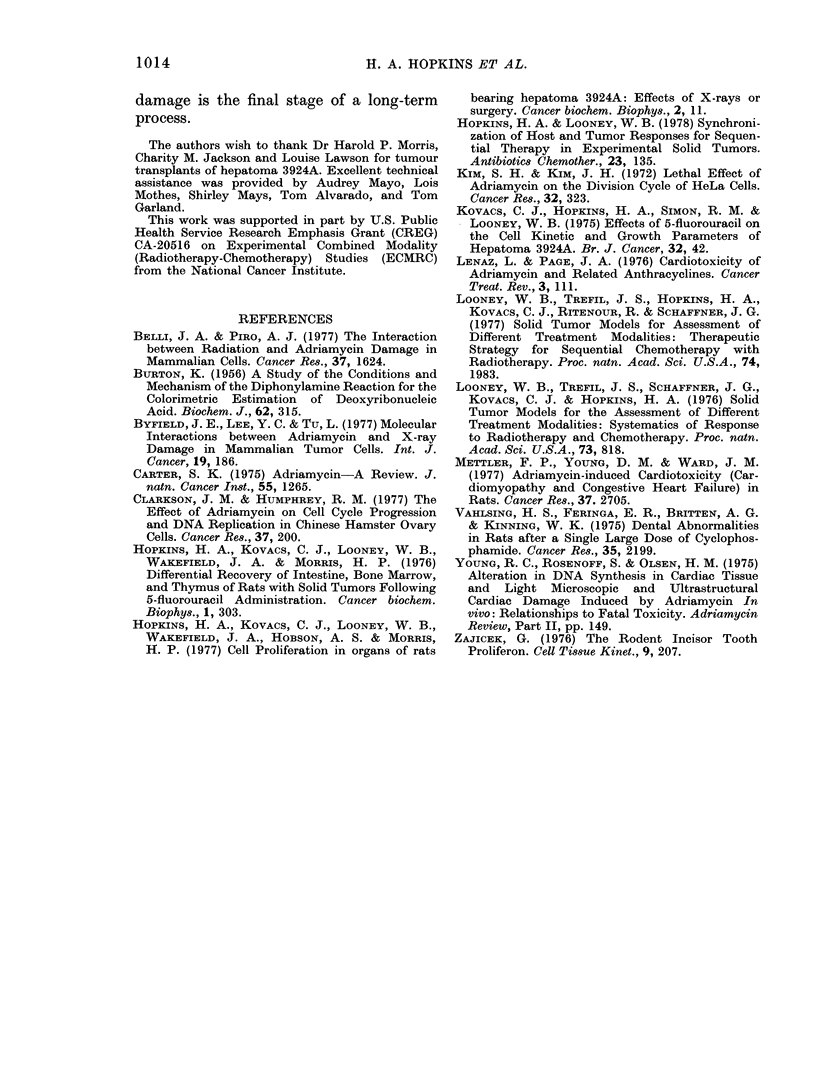# Response kinetics of host and experimental solid tumour after adriamycin.

**DOI:** 10.1038/bjc.1978.146

**Published:** 1978-06

**Authors:** H. A. Hopkins, W. B. Looney, K. Teja, A. S. Hobson, M. S. MacLeod

## Abstract

The effects of adriamycin (Adr) on the solid-tumour model, hepatoma 3924A, and on critical organs of the host, were determined at intervals after single injections of 60 mg/m2 of the agent. A reduced rate of tumour growth was evident 4 days after treatment, continued to Day 11, and then returned to rates comparable to control values. On Day 11 tumour volumes of treated animals were 38% of control. During the period of reduced growth, 3H-TdR incorporation into tumour DNA and percentage labelled tumour cells were less than control values. DNA concentration in tumour was not affected by drug treatment, which differs from observations made in other studies employing 5-fluorouracil (FU). No evidence of significantly increased necrosis or fibrosis of the tumour was found after Adr. The Adr treatment caused loss of 60% of the tibial marrow by Day 4, as measured by total DNA content. Marrow DNA recovered to control levels between Days 7 and 11. Incorporation of 3H-TdR into heart DNA was reduced more than 40% during the first week after Adr treatment; enhanced incorporation was observed on Day 11, and control levels were attained by Day 17. No significant pathological evidence of cardiac toxicity was found 2-21 days after Adr but degeneration of myocardial cells and oedema was prominent at 14 weeks.


*Br. J. Cancer* (1978) **37,** 1006

### RESPONSE KINETICS OF HOST AND EXPERIMENTAL SOLID TUMOUR AFTER ADRIAMYCIN

H. A. HOPKINS*†, W. B. LOONEY†, K. TEJA‡, A. S. HOBSON‡ AND M. S. MACLEOD†

*From the †Division of Radiobiology and Biophysics and the ‡Department of Pathology University of Virginia School of Medicine, Charlottesville, Virginia 22901, U.S.A.*

Received 17 January 1978 Accepted 22 February 1978

**Summary.—The effects of adriamycin (Adr) on the solid-tumour model, hepatoma 3924A, and on critical organs of the host, were determined at intervals after single injections of 60 mg/m$^2$ of the agent. A reduced rate of tumour growth was evident 4 days after treatment, continued to Day 11, and then returned to rates comparable to control values. On Day 11 tumour volumes of treated animals were 38% of control. During the period of reduced growth, $^3$H-TdR incorporation into tumour DNA and percentage labelled tumour cells were less than control values. DNA concentration in tumour was not affected by drug treatment, which differs from observations made in other studies employing 5-fluorouracil (FU). No evidence of significantly increased necrosis or fibrosis of the tumour was found after Adr. The Adr treatment caused loss of 60% of the tibial marrow by Day 4, as measured by total DNA content. Marrow DNA recovered to control levels between Days 7 and 11. Incorporation of $^3$H-TdR into heart DNA was reduced more than 40% during the first week after Adr treatment; enhanced incorporation was observed on Day 11, and control levels were attained by Day 17. No significant pathological evidence of cardiac toxicity was found 2–21 days after Adr but degeneration of myocardial cells and oedema was prominent at 14 weeks.**

THE kinetics of recovery for tumour and host organs after treatment with 5-fluorouracil (FU) have recently been determined for the experimental solid-tumour system, hepatoma 3924A (Kovacs *et al.*, 1975; Hopkins *et al.*, 1976; Looney *et al.*, 1976) and these data have been used in the design of an effective chemotherapy–radiotherapy combination for this tumour (Looney *et al.*, 1977). The present report summarizes data obtained for hepatoma 3924A and its host the ACI rat after treatment with adriamycin (Adr), a relatively new chemotherapeutic agent which is effective against a wide range of human neoplasms (Carter, 1975). Evaluation of the effects of Adr has included determinations of changes in tumour volume, changes in DNA content and $^3$H-TdR incorporation for marrow, intestinal mucosa, heart and tumour, tumour TdR labelling indices and pathological evaluations of tumour and host organs. Haematological data were obtained for peripheral blood and, in other studies, liver-function tests were performed, to obtain as much clinically relevant information as possible in this evaluation of the effects of Adr on both the solid tumour and its host.

### METHODS

*Animals and tumours.*—Inbred female ACI-strain rats (Laboratory Supply Co., Indianapolis, Ind. and Mammalian Genetics and Animal Production Section, National Cancer Institute) usually weighing 120–140 g were used. Tumour-bearing animals had s.c.

* To whom correspondence should be addressed at Box 392, University of Virginia Hospital, Charlottesville, VA 22901, U.S.A.

transplants of Morris hepatoma 3924A. The rats were caged individually in an air-conditioned room lighted from 8 a.m. to 8 p.m. and provided rat chow (Charles River Laboratories, Wilmington, Mass.) and water *ad libitum*.

*Drug.*—Adriamycin (Adr, synonym doxorubicin) was supplied by the Drug Synthesis & Chemistry Branch, Division of Cancer Treatment, National Cancer Institute. It was dissolved in 0·9% NaCl and usually administered by i.p. injection of doses of 60 mg/m$^2$ (10 mg/kg). Where i.v. or s.c. injections or differing doses of drug were given, these are indicated in the text.

*Experimental.*—At various times after injection of 60 mg/m$^2$ Adr and 1 h prior to killing, groups of 3 rats each were injected i.p. with 50 $\mu$Ci thymidine-(methyl)-$^3$H (sp. act. 3 Ci/mmol). At killing, blood for WBC, haematocrit and haemoglobin determinations was taken by cardiac puncture under ether anaesthesia. The tumour, tibia, heart and a 4 cm length of intestine immediately distal to the pylorus were removed for biochemical and histologic evaluations. Tissue specimens were routinely fixed in neutral formalin, embedded in paraffin, sectioned, and stained with haematoxylin and eosin. In addition, Masson stain was used for tumour. Autoradiographs were prepared with Kodak NTB-2 after Feulgen staining. Slides were exposed for 3 weeks, then developed with Kodak D-19.

The marrow was aspirated from the tibia with cold 0·9% NaCl. The 4 cm length of intestine was slit longitudinally and the mucosa stripped from the underlying muscle. Tumour and heart were chilled in cold 0·9% NaCl, weighed, and portions taken for determination of DNA and $^3$H counts in the DNA fractions. RNA was eliminated from all samples by alkaline hydrolysis followed by washing with cold 10% trichloroacetic acid. DNA was extracted by heating at 90°C for 20 min with 5% trichloroacetic acid and was measured by the method of Burton (1956). Calf thymus DNA (Sigma, St Louis, MO) was the standard. Radioactivity in the nucleic acid extracts was measured on a Beckman liquid scintillation spectrophotometer with external standardization.

Acute animal survival was determined 21 days after Adr injection or, if more than one dose was given, 21 days after the final injection.

### RESULTS

### *Changes in tumour growth and kinetics*

Treatment of rats bearing hepatoma 3924A with 60 mg/m$^2$ Adr as a single dose resulted in a reduced rate of growth for the tumour (Fig. 1) which was evident 4 days after treatment. The rate of tumour

FIG. 1.—Mean tumour volumes for hepatoma 3924A after Adr treatment. Each point is the mean (±s.e.) for 6 treated rats or 8 control rats. ●, 60 mg/m$^2$ Adr; ○, control.

growth was reduced until about Day 11 after treatment, at which time the rate of growth for treated tumours returned to control rates. Tumour volumes were 38% of control at that time. The time necessary to reach an average tumour volume of 5 cm$^3$ from initial volumes of 0·27 cm$^3$ was increased from 9 days for control to 14 days. During the period of reduced tumour growth, 4–11 days after treatment, incorporation of $^3$H-TdR into tumour DNA was inhibited relative to untreated tumours (Fig. 2) and percentage labelled interphase cells determined by autoradiography were similarly reduced. The concentration of DNA (mg DNA/g of tumour) was not altered by drug treatment (Fig. 2). Tumour sections stained with haematoxylin and eosin showed no effect of the drug on tumour histology or necrosis.

FIG. 2.—Effect of Adr on $^3$H-TdR incorporation into DNA, % labelled cells, and DNA concentration of hepatoma 3924A. Each point is the mean (±s.e.) for 3 rats, except 2 rats where standard error is not indicated. ●, 60 mg/m$^2$ Adr; ○, control.

FIG. 3.—Tibial marrow DNA after Adr treatment of ACI rats with and without tumours. Open symbols, no drug. Closed symbols, 60 mg/m$^2$ Adr. Squares, rats without tumours; Circles, rats with hepatoma 3924A.

Masson stain also did not indicate any significant increase of fibrosis within the tumour.

*Marrow*

There was loss of marrow after administration of Adr, amounting to ~60% by Day 4 as measured by mg DNA per tibial marrow (Fig. 3). Total DNA per tibial marrow is proportional to the total number of nucleated cells present at this marrow site, therefore it can be used as an index of marrow cellularity. Recovery to untreated control levels took place between Days 7 and 11. For rats with tumours the increases in marrow DNA content for the untreated rats, and after Day 14 for the treated rats, are the result of an increased granulocytopoiesis associated with the growth of hepatoma 3924A (Hopkins *et al.*, 1977). Neither the magnitude of the loss of marrow after Adr nor the timing of the recovery was appreciably affected by the presence of the tumour. Incorporation of $^3$H-TdR into marrow DNA was determined at intervals after drug administration, and is expressed in Fig. 4 (top) as d/min/mg DNA. The large apparent increases in specific activity of marrow DNA 2 to 4 days after Adr, possibly result from loss of marrow DNA (Fig. 3) rather than from increased DNA synthesis. Expressed as d/min/tibia (Fig. 4, bottom), $^3$H-TdR incorporation is constant but depressed relative to control values, during the period of time that marrow is being lost from the tibia. It is remarkable that recovery of marrow DNA content occurs without an increase in $^3$H-precursor incorporation. This differs from the situation observed after treatment of rats with FU (Hopkins *et al.*, 1976). At longer times after 60 mg/m$^2$ Adr both DNA content and specific activity of the DNA were depressed 25% or more relative to control (Table I).

*Haematological data*

Changes in WBC concentrations in peripheral blood after the administration

FIG. 4.— $^3$H-TdR incorporation into tibial marrow DNA after Adr treatment, expressed as d/min/mg DNA and as d/min/tibial marrow. ●, 60 mg/m$^2$ Adr; ○, control.

of Adr to animals with tumours are shown in Table II. WBC concentrations were depressed 30–40% below initial control values on Days 4, 7 and 11 after treatment. Hepatoma 3924A stimulates granulocytopoiesis in the ACI rat (Hopkins *et al.*, 1977) accounting for the increasing WBC values for the controls and for treated animals after Day 11. Haemoglobin and haematocrit values are not shown, but were similar for drug-treated and control rats, with the greatest difference, observed on Day 7, being only 18%.

*Intestinal mucosa*

DNA measurements revealed that the cellularity of the intestinal mucosa after i.p. injection of Adr was ~20% below control level for 4 days, (Fig. 5A). Incorporation of $^3$H-TdR into DNA was less than control at 24 h after treatment, then increased markedly to more than twice control values on Day 4 (Fig. 5B). DNA accumulated after the episode of increased $^3$H-TdR incorporation, and remained elevated for the duration of the experi-

TABLE I.—*Effect of Route of Administration of 60 mg/m$^2$ Adr on $^3$H-TdR Incorporation and DNA Content of Tibial Marrow, Heart and Intestinal Mucosa of Rats*

| Post-treatment interval (weeks) | Route of administration i.v. | s.c. | i.p. | Control |
|---|---|---|---|---|
| | mg DNA/Tibial Marrow | | | |
| 3 | 0·40±0·06 | 0·42±0·02 | 0·50 (2 rats) | 0·42±0·05 |
| 8 | 0·38±0·06 | 0·28±0·01 | 0·49±0·12 | 0·38±0·02 |
| 12 | 0·31±0·02 | 0·30±0·03 | dead | 0·39±0·04 |
| | d/min/mg Marrow DNA × 10$^{-5}$ | | | |
| 3 | 1·20 (2 rats) | 1·97±0·55 | 1·98 (2 rats) | 2·02±0·08 |
| 8 | 1·26±0·05 | 1·48±0·07 | 0·78±0·22 | 2·03±·011 |
| 12 | 0·91±0·04 | 1·41±0·34 | dead | 2·01±0·15 |
| | mg DNA/4 cm Intestinal Mucosa | | | |
| 3 | 1·14±0·32 | 1·39±0·20 | 1·62 (2 rats) | 1·42±0·11 |
| 8 | 1·35±0·11 | 1·44±0·08 | 1·70±0·45 | 1·39±0·06 |
| 12 | 1·35±0·10 | 1·26±0·09 | dead | 1·27±0·04 |
| | d/min/mg Mucosal DNA × 10$^{-5}$ | | | |
| 3 | 2·16 (2 rats) | 3·45±0·62 | 3·80 (2 rats) | 3·58±0·14 |
| 8 | 3·20±0·31 | 2·70±0·32 | 1·81±0·70 | 3·11±0·19 |
| 12 | 2·15±0·15 | 2·98±0·49 | dead | 3·46±0·26 |
| | d/min/mg Heart DNA × 10$^{-4}$ | | | |
| 3 | 1·77±0·15 | 1·65±0·31 | 1·80±0·15 | 0·72±0·11 |
| 8 | 0·49±0·08 | 1·60±0·30 | 1·62±0·66 | 0·95±0·11 |
| 12 | 1·36±0·29 | 1·02±0·31 | dead | 0·78±0·03 |

3 animals in each group unless otherwise stated.
Figures are means ± s.e.

TABLE II.—*Changes in Peripheral WBC Counts of ACI Rats with Hepatoma 3924A after Adr Administration*

| Time after Adr (days) | WBC counts/mm³ Controls | Treated |
|---|---|---|
| 2 | 4625± 463* | 4800 (2 rats) |
| 4 | 4600± 183 | 2833± 219 |
| 7 | 5275± 397 | 3050 (2 rats) |
| 9 | 7350± 796 | 5500± 643 |
| 11 | 6775± 912 | 3333± 384 |
| 13 | 7725± 364 | 6600± 929 |
| 15 | 8550±1041 | 7567±1613 |
| 17 | 9775± 669 | 8667± 437 |
| 21 | 9367± 484 | 7033±1073 |

* Mean±s.e.—4 animals in each Control Group and 3 animals in each Treated Group (unless otherwise stated).

ment. However, the increased cellularity of the intestinal mucosa after Adr treatment appears to be related to the i.p. route of administration. I.v. or s.c. injections do not increase the DNA content of the mucosa at 3, 8 or 12 weeks after the drug (Table I).

FIG. 5.—Effect of Adr on intestinal mucosa. A. DNA content. B. TdR incorporation. ●, 60 mg/m² Adr; ○, control.

*Heart*

In heart, inhibition of $^3$H-TdR incorporation into DNA, by more than 40%, was observed during the first week after Adr was given (Fig. 6A). Enhanced incorporation was seen on Day 11, with return to control levels by Day 17. Average heart DNA content for drug-treated rats was not different from control, after adjusting for differences in animal weights (Fig. 6B). Rats injected with 60 mg/m² Adr by i.v., s.c., or i.p. routes and killed 3, 8 or 12 weeks later, often showed increased $^3$H-TdR incorporation in heart DNA (Table I). Incorporation was about twice that of control rats at these times.

Foci of myocardial-cell degeneration accompanied by convergence of phagocytic cells were occasionally seen in the histologic preparations from rats treated 2 to 21 days earlier with 60 mg/m² Adr, and in control animals as well. However, in rats surviving this drug treatment for 14 weeks, the incidence of these pathological changes was much increased.

FIG. 6.—Effect of Adr on heart DNA. A. TdR incorporation into heart DNA. B. Mean DNA content of heart. Solid symbols, 60 mg/m² Adr; open symbols, control.

FIG. 7.—Survival of rats given multiple injections of Adr as a function of the interval between injections. Each group contained 10 rats.

Oedema and degeneration of myocardial fibres were prominent. Some intracytoplasmic vacuoles were also noted. The degree of damage to the myocardium was dose related, being less severe for rats given 3 injections of 30 or 45 mg/m$^2$ at 14 day intervals than for those given 60 mg/m$^2$ on this intermittent schedule.

*Animal survival*

The relationship between animal survival and the interval separating successive doses of Adr was examined in tumour-free rats and these data are shown in Fig. 7. The i.p. route was utilized for this experiment, and survival was determined 21 days after the last injection. All rats survived a single 60 mg/m$^2$ dose of Adr. Two 60 mg/m$^2$ doses given together were uniformly fatal. Intervals between 2 injections of 4 and 8 days gave increasing survivals. Deaths were most frequent during the 4-6-day period immediately after the second injection. Fluid accumulation in both the pleural and peritoneal cavities was commonly seen at autopsy. When the interval was 12 or 16 days, 80% of the rats were alive 21 days after the second injection. Three 60 mg/m$^2$ injections at 14-day intervals gave a survival of 60%, which improved to 100% when the individual doses were reduced to 45 or 30 mg/m$^2$.

Although TdR incorporation remained suppressed during recovery of marrow DNA content after treatment with Adr (Fig. 4B) injection of the proliferation-dependent agent FU during this recovery period proved fatal. For doses of 900 mg/m$^2$ (150 mg/kg) FU at 7 or 11-day intervals after 60 mg/m$^2$ Adr, 21-day survival was 0 and 70%, respectively. Simultaneous administration of the 2 agents gave a survival of 60%. Total tibial DNA, not $^3$H-TdR incorporation, is the valid indicator of tolerance for a second treatment when Adr is the first agent.

Intestinal adhesions occur after Adr is given i.p. However, this does not compromise the validity of the acute-survival data in Fig. 7 since deaths related to intestinal adhesions begin to occur only after 6 weeks. These deaths can be avoided by using the i.v. or s.c. routes.

Another complication which can affect long-term toxicity data, and is independent of route of administration, is an effect of cytotoxic drugs on the continuously growing rodent incisor teeth. Zajicek (1976) has described the rodent incisor-tooth proliferon as the site of 5 cell populations proliferating in harmony. Drugs such as cyclophosphamide (Vahlsing, 1975), FU (Hopkins, unpublished) and Adr perturb these populations, with the result that the incisors become overgrown and the rat cannot eat a pelleted diet. Clipping of the teeth and feeding powdered diet will prevent death from starvation. It is important that animal mortality from overgrown teeth be carefully eliminated from toxicity data since this is not a factor in the clinical use of these drugs.

### DISCUSSION

Adriamycin binds to DNA, and this is believed to be responsible for its cytotoxic effects, although the exact mechanism remains unknown. Synthesis of both RNA

and DNA is inhibited by the drug for *in vitro* cell cultures (Kim and Kim, 1972) and more recently, single and double DNA-strand breaks have been seen after treatment of intact cells with Adr (Byfield *et al.*, 1977). Others have noted the similar, but not identical, effects of Adr and radiation on cells (Belli and Piro, 1977) and the possible involvement of ribosomal RNA synthesis (Clarkson and Humphrey, 1977).

The changes in tumour morphology and quantitative changes in tumour kinetics after Adr described here may be compared with changes in the same solid-tumour model after an equally effective dose of the proliferation-dependent cancer-chemotherapeutic agent FU. In studies not detailed here, we found no significant differences in mean tumour volumes of animals given 900 mg/m$^2$ (150 mg/kg) FU or 60 mg/m$^2$ Adr over a period of 16 days after treatment. The dose of Adr used in the present study is therefore equivalent to that of 5-FU (900 mg/m$^2$) in previous studies, with respect to total perturbation of tumour growth. The TdR labelling index was above control values 2 days after Adr administration. This returned to control values by Day 4 and remained below the control unitl Day 16. The rate of DNA synthesis was also elevated on Day 2 but depressed from Day 4 until Day 16. The elevation of both the DNA specific activity and labelling index on Day 2 suggests a redistribution of cells within the cell cycle, with an increased proportion of cells in the "S" period, as occurs with FU (Kovacs *et al.*, 1975). The depression of both DNA specific activity and labelling indices between Day 4 and Day 16 could be the result of the direct effects of Adr on DNA synthesis, as well as a reduction of S-phase cells. Clarkson and Humphrey (1977) reported that Chinese hamster ovary cells treated in mid-S phase showed a dose-dependent progression delay which was also reflected in the rates of DNA replication in the subsequent S phase. For hepatoma 3924A, the percentage of labelled cells remain high up to 1 week after FU administration (Kovacs *et al.*, 1975).

There are 2 other major changes which occur after FU which were virtually absent in the Adr treated tumours: within 48 h after treatment with 900 mg/m$^2$ FU, there was a gradual decrease in DNA concentration of the treated tumours. By 7–8 days after treatment, DNA concentration reached a nadir, and gradual restoration to the DNA concentration observed for untreated tumours occurred over the next 2-week period. This reduction of DNA/g tumour to 70% of that for untreated tumours reflects cell death and eventual removal of dead cells beyond the cell loss normal for growing tumours. Since protein/g tumour was not affected by treatment with FU, changes in water content cannot be responsible for the decrease in DNA concentration. The DNA concentration in the Adr-treated tumours remained unchanged over the entire 22-day period of the study.

The tissue composition of untreated tumours remains relatively constant, at 51% tumour, 18% necrotic, 26% connective and 5% blood, for the range of tumour volumes used in both the FU and Adr studies. Tissue composition after treatment with 900 mg/m$^2$ FU, showed that the relative number of tumour cells were reduced to a minimum of 50% of untreated-tumour values 48 h after treatment. Concomitantly, increases in both necrotic and connective tissue were found over this 48 h period. The relative tissue composition was restored to untreated values by 7 days. Little or no pathological changes were observed in the tumour during this period after Adr.

The lack of pathological changes and changes in DNA concentration in the tumours after Adr indicates that the cellular responses are more gradual than with FU. The results of the 2 studies suggest that the proliferation-dependent agent FU causes an abrupt and well-defined sequence of changes in tumour morphology and kinetics, whereas Adr fails to elicit these abrupt and welldefined changes. However, the end results on the perturbations of tumour growth and the reduction in tumour volume 11 days after the 2 agents are comparable.

The depressed peripheral WBC counts after 60 mg/m$^2$ Adr were 60–70% of controls, compared to 50% of controls for the 900 mg/m$^2$ dose of FU (Hopkins and Looney, 1978). The total tibial DNA content has been used as an index of marrow reserve. There was a loss of 60% of the marrow after 60 mg/m$^2$ of Adr and of 90% after 900 mg/m$^2$ of FU. This dose of FU which produces an effect on tumour growth comparable to 60 mg/m$^2$ Adr, causes a much greater depression of the marrow. However, the more pronounced effect of FU upon marrow is not reflected in the decrease in peripheral WBC counts.

The survival studies with 2 injections of Adr separated by various intervals of time were comparable to survival studies after FU. Both studies show that giving the second dose of either agent prior to 11 days results in varying degrees of animal mortality. Giving the second dose of either agent 11 days or more after the first allows the animal to survive. These comparable recovery kinetics after 2 cancer-chemotherapy agents with different mechanisms of action, indicate that this phenomenon is primarily related to the recovery kinetics of the haematopoietic system. The recovery of the gastrointestinal mucosa occurs prior to marrow for either drug. These studies indicate that recovery of the haematopoietic system is one factor to be considered in the design of protocols using Adr.

On the basis of both clinical and experimental studies (Lenaz and Page, 1976) the heart is considered to be the dose-limiting organ for accumulated toxicity for Adr. Cardiac dysfunction appears to be a factor in the early deaths reported here after a single dose of Adr of 120 mg/m$^2$ or 2 divided doses of 60 mg/m$^2$ given 4 or 8 days after the first. Accumulation of fluid was seen in both the pleural and peritoneal cavities at autopsy, suggesting congestive heart failure. However, no specific pathological changes were noted in hearts of animals given 60 mg/m$^2$ of Adr 2–21 days prior to sacrifice. Studies over a 14-week period demonstrated cardiac damage similar to that seen by others in the rat (Mettler *et al.*, 1977) and other species. Vacuoles within mycoardial cells and oedema were prominent after this long interval. In addition, non-specific changes such as foci of myocardial cell degeneration, occasionally seen in both treated and control rats 2–21 days from start of experiment, were more numerous and prominent in the 14-week animals.

The biochemical results appear to substantiate the pathological findings of slowly increasing cardiac damage over long periods of time. The increased incorporation of DNA precursors into heart DNA is believed to occur mostly in the endothelial cells of the capillaries and in the mitochondria of myocardal fibres (Lenaz and Page, 1976). Adr induces extensive damage to DNA in cell cultures (Byfield *et al.*, 1977). The continuous and long-term increased specific activity in heart DNA could also be related to repair of this damage. These studies on depression of DNA synthesis in the rat after a large single dose of Adr are comparable to the findings in the mouse (Young *et al.*, 1975). The maximum depression of DNA-synthesis occurs between 3 and 6 days in both the rat and mouse, with the DNA synthetic rate returning to control level by Day 9 in the rat. Recovery data are not available for the mouse. The rate of DNA synthesis in the rat heart after both FU (900 mg /m$^2$) and Adr (60 mg/m$^2$) increased to values above controls on Day 11 but returned to control levels by Day 18. The long-term studies showed elevated rates of heart DNA synthesis in rats 3, 8 and 12 weeks after a large single dose of Adr (60 mg /m$^2$) whether i.v., s.c., or i.p. (Table I). If this long-term increase in specific activity of heart DNA is associated with repair, it would reinforce the pathological observation that cardiac damage is the final stage of a long-term process.

The authors wish to thank Dr Harold P. Morris, Charity M. Jackson and Louise Lawson for tumour transplants of hepatoma 3924A. Excellent technical assistance was provided by Audrey Mayo, Lois Mothes, Shirley Mays, Tom Alvarado, and Tom Garland.

This work was supported in part by U.S. Public Health Service Research Emphasis Grant (CREG) CA-20516 on Experimental Combined Modality (Radiotherapy-Chemotherapy) Studies (ECMRC) from the National Cancer Institute.

### REFERENCES

BELLI, J. A. & PIRO, A. J. (1977) The Interaction between Radiation and Adriamycin Damage in Mammalian Cells. *Cancer Res.*, **37**, 1624.

BURTON, K. (1956) A Study of the Conditions and Mechanism of the Diphonylamine Reaction for the Colorimetric Estimation of Deoxyribonucleic Acid. *Biochem. J.*, **62**, 315.

BYFIELD, J. E., LEE, Y. C. & TU, L. (1977) Molecular Interactions between Adriamycin and X-ray Damage in Mammalian Tumor Cells. *Int. J. Cancer*, **19**, 186.

CARTER, S. K. (1975) Adriamycin—A Review. *J. natn. Cancer Inst.*, **55**, 1265.

CLARKSON, J. M. & HUMPHREY, R. M. (1977) The Effect of Adriamycin on Cell Cycle Progression and DNA Replication in Chinese Hamster Ovary Cells. *Cancer Res.*, **37**, 200.

HOPKINS, H. A., KOVACS, C. J., LOONEY, W. B., WAKEFIELD, J. A. & MORRIS, H. P. (1976) Differential Recovery of Intestine, Bone Marrow, and Thymus of Rats with Solid Tumors Following 5-fluorouracil Administration. *Cancer biochem. Biophys.*, **1**, 303.

HOPKINS, H. A., KOVACS, C. J., LOONEY, W. B., WAKEFIELD, J. A., HOBSON, A. S. & MORRIS, H. P. (1977) Cell Proliferation in organs of rats bearing hepatoma 3924A: Effects of X-rays or surgery. *Cancer biochem. Biophys.*, **2**, 11.

HOPKINS, H. A. & LOONEY, W. B. (1978) Synchronization of Host and Tumor Responses for Sequential Therapy in Experimental Solid Tumors. *Antibiotics Chemother.*, **23**, 135.

KIM, S. H. & KIM, J. H. (1972) Lethal Effect of Adriamycin on the Division Cycle of HeLa Cells. *Cancer Res.*, **32**, 323.

KOVACS, C. J., HOPKINS, H. A., SIMON, R. M. & LOONEY, W. B. (1975) Effects of 5-fluorouracil on the Cell Kinetic and Growth Parameters of Hepatoma 3924A. *Br. J. Cancer*, **32**, 42.

LENAZ, L. & PAGE, J. A. (1976) Cardiotoxicity of Adriamycin and Related Anthracyclines. *Cancer Treat. Rev.*, **3**, 111.

LOONEY, W. B., TREFIL, J. S., HOPKINS, H. A., KOVACS, C. J., RITENOUR, R. & SCHAFFNER, J. G. (1977) Solid Tumor Models for Assessment of Different Treatment Modalities: Therapeutic Strategy for Sequential Chemotherapy with Radiotherapy. *Proc. natn. Acad. Sci. U.S.A.*, **74**, 1983.

LOONEY, W. B., TREFIL, J. S., SCHAFFNER, J. G., KOVACS, C. J. & HOPKINS, H. A. (1976) Solid Tumor Models for the Assessment of Different Treatment Modalities: Systematics of Response to Radiotherapy and Chemotherapy. *Proc. natn. Acad. Sci. U.S.A.*, **73**, 818.

METTLER, F. P., YOUNG, D. M. & WARD, J. M. (1977) Adriamycin-induced Cardiotoxicity (Cardiomyopathy and Congestive Heart Failure) in Rats. *Cancer Res.*, **37**. 2705.

VAHLSING, H. S., FERINGA, E. R., BRITTEN, A. G. & KINNING, W. K. (1975) Dental Abnormalities in Rats after a Single Large Dose of Cyclophosphamide. *Cancer Res.*, **35**, 2199.

YOUNG, R. C., ROSENOFF, S. & OLSEN, H. M. (1975) Alteration in DNA Synthesis in Cardiac Tissue and Light Microscopic and Ultrastructural Cardiac Damage Induced by Adriamycin *In vivo*: Relationships to Fatal Toxicity. *Adriamycin Review*, Part II, pp. 149.

ZAJICEK, G. (1976) The Rodent Incisor Tooth Proliferon. *Cell Tissue Kinet.*, **9**, 207.